# Phosphonate-substituted zirconium oxo clusters

**DOI:** 10.1007/s00706-015-1519-3

**Published:** 2015-07-02

**Authors:** Matthias Czakler, Ulrich Schubert

**Affiliations:** Institute of Materials Chemistry, Vienna University of Technology, Vienna, Austria

**Keywords:** Zirconium alkoxides, Phosphonate ligands, Structure analysis

## Abstract

**Abstract:**

The phosphonate-substituted zirconium oxo clusters Zr_6_O_2_(OBu)_12_(O_3_PPh)_4_ and Zr_7_O_2_(O*i*Pr)_12_(O_3_PCH_2_CH_2_CH_2_Br)_6_, with octahedrally coordinated Zr atoms, were synthesized by reaction of zirconium alkoxides with phosphonic acid bis(trimethylsilyl) esters. The basic structural motif are Zr_3_O(µ_2_-OR)_3_(OR)_3_ units which are connected in different ways.

**Graphical abstract:**

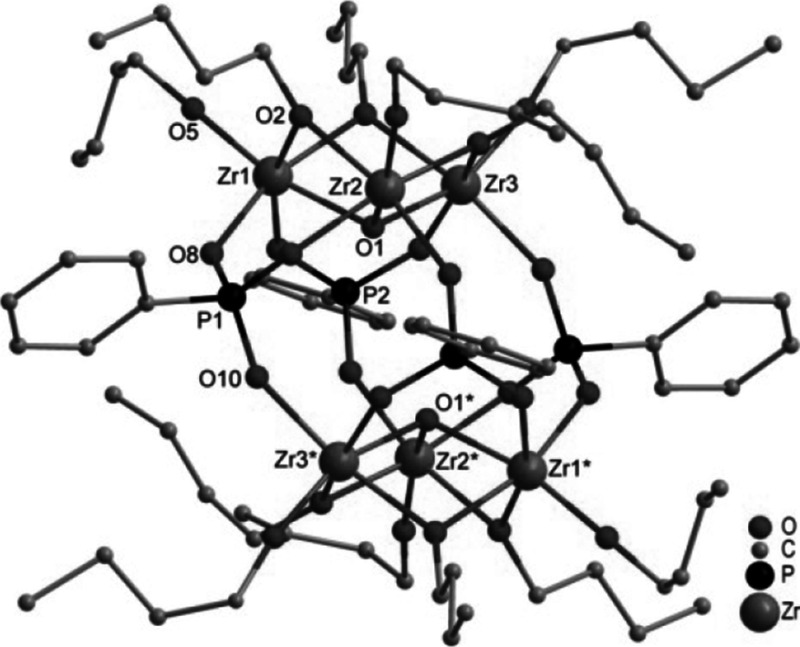

## Introduction

Many phosphonate-substituted zirconium compounds with 1D, layered 2D, and 3D interconnected structures have been reported [1–3], including coordination polymers with bis- or tetra-phosphonate ligands [4–11]. Surprisingly, no zirconium oxo clusters with phosphonate ligands are known, although zirconium oxo clusters with a variety of other bi- or multidentate ligands have been prepared and zirconia nanoparticles are frequently stabilized by phosphonate groups [12, 13]. In this article, we report the preparation and structural characterization of the first phosphonate-substituted zirconium oxo clusters.

Phosphonate-substituted metal compounds are commonly prepared from the corresponding phosphonic acids or their metal salts. We have recently shown that titanium oxo clusters can be easily obtained from the reaction of titanium alkoxides with phosphonic acid bis(trimethylsilyl) esters [14, 15]. The esters have the advantage of being soluble in organic solvents. Their reaction with alcohol added to the reaction mixture liberates phosphonic acid which substitutes part of the OR groups of Ti(OR)_4_ in a relatively fast reaction. Oxo groups are generated in situ either by water originating from esterification of (coordinated or non-coordinated) phosphonic acid or by non-hydrolytic processes.

## Results and discussion

**Fig. 1:**
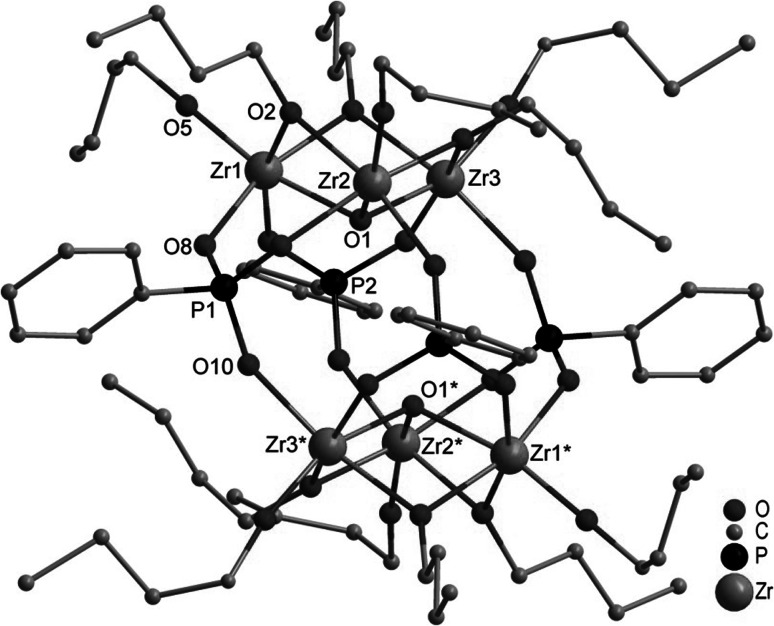
Molecular structure of Zr_6_(*μ*_3_-O)_2_(*μ*_2_-OBu)_6_(OBu)_6_(O_3_PPh)_4_ (**1**). Hydrogen atoms are omitted for clarity. Selected bond lengths/pm and angles/°: Zr(1)-O(1) 211.6(5), Zr(1)-O(2) 214.8(5), Zr(1)-O(3) 214.5(5), Zr(1)-O(5) 192.9(5), Zr(1)-O(8) 209.2(5), Zr(1)-O(11) 209.9(5), Zr(2)-O(1) 208.1(5), Zr(2)-O(2) 215.8(5), Zr(2)-O(4) 215.5(8), Zr(2)-O(6) 192.6(5), Zr(2)-O(9) 210.4(5), Zr(2)-O(13) 207.7(5), Zr(3)-O(1) 208.7(5), Zr(3)-O(3) 215.3(5), Zr(3)-O(4) 215.3(9), Zr(3)-O(7) 193.4(5), Zr(3)-O(10) 209.2(5), Zr(3)-O(12) 209.4(5); Zr(1)-O(1)-Zr(2) 106.1(2), Zr(1)-O(1)-Zr(3) 106.0(2), Zr(2)-O(1)-Zr(3) 109.6(2), Zr(1)-O(2)-Zr(2) 102.3(2), Zr(1)-O(3)-Zr(3) 102.7(2), Zr(2)-O(4)-Zr(3) 104.5(4)

Crystals of Zr_6_O_2_(OBu)_12_(O_3_PPh)_4_ (**1**, Fig. 1) were obtained when Zr(OBu)_4_ was reacted with bis(trimethylsilyl)phenylphosphonate in a 2:1 ratio.

The basic structural motif in centrosymmetric **1** are two Zr_3_O(*μ*_2_-OBu)_3_(OBu)_3_ moieties (Zr_3_O). They are interconnected with each other through four bridging phenylphosphonate ligands which are arranged up, up, down, down. Each phosphonate ligand binds to two zirconium atoms of one Zr_3_O triangle and one zirconium atom of the other Zr_3_O triangle and is, therefore, coordinating 3.111 (w.xyz refers to the number of metal atoms to which the phosphonate ligand is coordinated [w], and the number of metal atoms to which each oxygen is coordinated [x, y, z] [16]). The crystallographic symmetry of **1** is retained in solution since the ^31^P NMR spectrum in CD_2_Cl_2_ showed only one signal at 6.57 ppm. The expected number of signals with the expected shifts was observed in the ^1^H NMR spectrum.

The most surprising feature of **1** is that all zirconium atoms are octahedrally coordinated. This is remarkable since higher coordination numbers (7–9) are mostly found in zirconium oxo clusters. The structure of **1** is different from that of oxo clusters obtained from reactions of Ti(O*i*Pr)_4_ with bis(trimethylsilyl) phosphonates although Ti is also six-coordinated there. M_3_O(*μ*_2_-OR)_3_(OR)_3_ units are the basic structural motif in both cases. While two Zr_3_O units are directly connected with each other in **1**, the two Ti_3_O units in Ti_7_O_2_(O*i*Pr)_12_(O_3_PR)_6_ (R=CH_2_CH_2_CH_2_Cl or benzyl) are connected through a central Ti atom [14]. In the case of titanium, structures Ti_4_(µ_3_-O)(µ_2_-O*i*Pr)_3_(O*i*Pr)_5_(O_3_PR)_3_L (L = neutral ligand) and dimers thereof were also obtained, where the Ti_3_O unit is capped by a Ti(O*i*Pr)_2_L group.

**Fig. 2:**
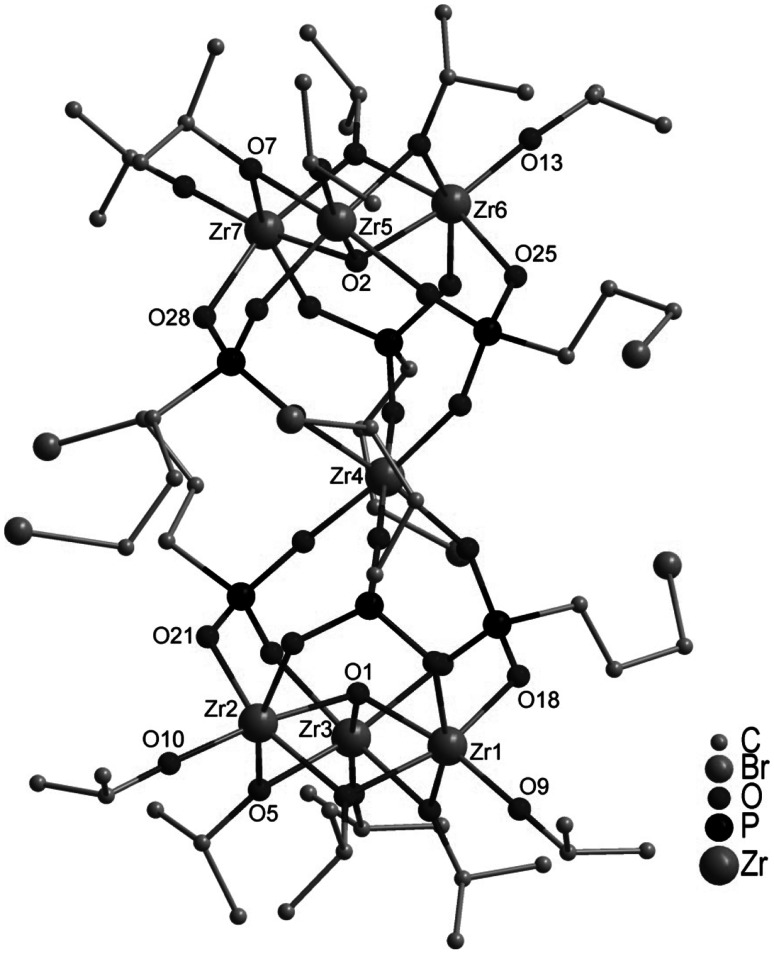
Molecular structure of Zr_7_O_2_(*µ*_2_-O*i*Pr)_6_(O*i*Pr)_6_(O_3_PCH_2_CH_2_CH_2_Br)_6_ (**2**). Hydrogen atoms are omitted for clarity. Selected bond lengths/pm and angles/°: O(1)-Zr(1) 207.9(4), O(1)-Zr(2) 209.3(4), O(1)-Zr(3) 208.2(4), O(2)-Zr(5) 208.9(4), O(2)-Zr(6) 209.2(4), O(2)-Zr(7) 208.8(4), O(5)-Zr(2) 216.4(4), O(5)-Zr(3) 217.0(4), O(7)-Zr(5) 218.6(4), O(7)-Zr(7) 216.5(5), O(9)-Zr(1) 194.2(5), O(10)-Zr(2) 193.1(5), O(13)-Zr(6) 192.2(5), O(18)-Zr(1) 210.1(4), O(21)-Zr(2) 211.7(4), O(23)-Zr(4) 206.0(4), O(25)-Zr(6) 211.1(4), O(26)-Zr(4) 207.4(4), O(28)-Zr(7) 210.6(5); Zr(1)-O(1)-Zr(2) 108.09(18), Zr(7)-O(7)-Zr(5) 101.9(2)

A zirconium oxo cluster isostructural to Ti_7_O_2_(O*i*Pr)_12_(O_3_PR)_6_, viz. Zr_7_O_2_(*µ*_2_-O*i*Pr)_6_(O*i*Pr)_6_(O_3_PCH_2_CH_2_CH_2_Br)_6_ (**2**, Fig. 2), was, however, obtained in another experiment, i.e., reaction of Zr(O*i*Pr)_4_ with bis(trimethyl)silyl(3-bromopropyl)phosphonate, methacrylic acid, and water. Since water generation by esterification of phosphonic acid (as in the first experiment) is relatively slow, water was deliberately added. Methacrylic acid was added anticipating an oxo cluster with a mixed ligand sphere as had been the case for analogous reactions with Ti(OR)_4_ [15, 17]. No mixed ligand cluster was obtained, however, in the reaction of Zr(O*i*Pr)_4_.

The symmetry of **2** is retained in solution as only one signal at 30.6 ppm was observed in the ^31^P NMR spectrum in C_6_D_6_. The ^1^H NMR spectrum shows only two doublets for the isopropoxo CH_3_ groups as well as two multiplets of the CH groups. Therefore, all terminal as well as all bridging isopropoxo ligands are symmetry related in solution.

## Conclusions

The coordination chemistry of titanium and zirconium, including that of metal oxo clusters, is usually quite different even if the same reaction conditions and stoichiometric ratios of the reactants are employed. This is due to the different coordination numbers.

The surprising outcome of the work reported in this article is that oxo clusters were obtained in the reaction of M(OR)_4_ (M = Ti, Zr) with bis(trimethyl)silylphosphonates where the coordination numbers and geometries of both Ti and Zr were the same. For this reason, the structures of the obtained Zr clusters were the same as those of Ti oxo clusters (for **2**) or very closely related (for **1**). A possible reason for this feature might be that the M_3_O(*μ*_2_-OR)_3_(OR)_3_ moiety appears to be a very robust building block, as already postulated earlier [14].

## Experimental

All operations were carried out in a moisture- and oxygen-free argon atmosphere using Schlenk techniques. 2-Propanol and 1-butanol were dried by distilling twice from sodium metal. The phosphonates were prepared as previously reported [14, 15]. Zirconium isopropoxide and zirconium *n*-butoxide were obtained from Sigma-Aldrich and used without further purification.

### *Zr*_*6*_*O*_*2*_*Cluster Zr*_*6*_*(μ*_*3*_-*O)*_*2*_*(μ*_*2*_-*OBu)*_*6*_*(OBu)*_*6*_*(O*_*3*_*PPh)*_*4*_ (**1**, C_72_H_128_O_26_P_4_Zr_6_)

Bis(trimethylsilyl) phenylphosphonate (100 mm^3^, 0.33 mmol) was added to 302 mm^3^ of Zr(OBu)_4_ (0.66 mmol) in 2 cm^3^ of BuOH. After 16 weeks at room temperature part of the solvent was removed from the clear solution. Crystals of **1** were obtained after 5 additional weeks at −20 °C. Yield 50 mg (22 %); ^1^H NMR (CD_2_Cl_2_, 250 MHz): *δ* = 0.61–1.07 (m, 36H, CH_3_), 1.08–1.86 (m, 48H, CH_2_), 3.50–4.38 (m, 24H, CH_2_O), 7.30–7.53 (m, 12H, CH), 7.68–8.05 (m, 8H, CH) ppm; ^13^C NMR (CD_2_Cl_2_, 62.9 MHz): *δ* = 13.65, 13.93 (CH_3_), 18.88, 19.10 (*C*H_2_CH_3_), 34.98, 35.60, 36.08 (*C*H_2_CH_2_O), 69.66, 69.92, 70.12 (CH_2_O), 127.58, 127.82, 130.87, 131.02 (CH) ppm; ^31^P NMR (CD_2_Cl_2_, 101.2 MHz): *δ* = 6.57 ppm.

### *Zr*_*7*_*O*_*2*_*Cluster Zr*_*7*_*O*_*2*_*(µ*_*2*_-*OiPr)*_*6*_*(OiPr)*_*6*_*(O*_*3*_*PCH*_*2*_*CH*_*2*_*CH*_*2*_*Br)*_*6*_ (**2**, C_54_H_120_Br_6_O_32_P_6_Zr_7_)

Methacrylic acid (33.8 mm^3^, 0.4 mmol) was added to a solution of 465 mg of Zr(O*i*Pr)_4_ (1.2 mmol) in 2 cm^3^ of 2-propanol followed by addition of 120 mm^3^ of bis(trimethyl)silyl(3-bromopropyl)phosphonate (0.4 mmol). After 5 min of vigorous stirring, 10.8 mm^3^ of water in 1 cm^3^ of 2-propanol were added quickly. Crystals of **2** were obtained after 2 weeks. Yield 20 mg (12 %); ^1^H NMR (C_6_D_6_, 250 MHz): *δ* = 1.37 (d ^3^*J*_H,H_ = 6.10 Hz, 36H, CH_3_), 1.59 (d, ^3^*J*_H,H_ = 6.24 Hz, 36H, CH_3_), 1.81–1.95 (m, 12H, CH_2_P), 2.28–2.41 (m, 12H, *CH*_*2*_CH_2_P), 3.57 (t, ^3^*J*_H,H_ = 6.55 Hz, CH_2_Br), 4.37 (m, 6H, CH), 4.97 (m, 6H, CH) ppm; ^31^P NMR (C_6_D_6_, 101.2 MHz): *δ* = 30.58 ppm.

### X-Ray structure analyses

All measurements were performed using Mo*K*_*α*_ radiation (*λ* = 71.073 pm). Data were collected on a Bruker AXS Smart Apex II four-circle diffractometer with *κ*-geometry at 100 K with *φ* and *ω*-scans and 0.5° frame width (Table 1). The data were corrected for polarization and Lorentz effects, and an empirical absorption correction (SADABS) was applied. The cell dimensions were refined with all unique reflections. Saint Plus software (Bruker Analytical X-ray Instruments, 2007) was used to integrate the frames. Symmetry was checked with the program PLATON.

**Table 1:** Crystal data and structure refinement details of **1** and **2**

Compound	**1**	**2**
Emp. formula	C_72_H_128_O_26_P_4_Zr_6_	C_54_H_120_Br_6_O_32_P_6_Zr_7_
*M*_*r*_	2080.94	2585.32
Crystal system	Triclinic	Triclinic
Space group	*P* $\bar{1}$	*P* $\bar{1}$
*a*/pm	1302.35(6)	1330.4(5)
*b*/pm	1332.92(6)	1885.7(8)
*c*/pm	1411.35(7)	2076.1(9)
*α*/°	70.525(3)	72.26(1)
*β*/°	81.574(3)	84.90(1)
*γ*/°	80.357(3)	70.27(1)
*V*/pm^3^ × 10^6^	2266.3(2)	4669(3)
*Z*	1	2
*D*_x_/g cm^−3^	1.525	1.839
*µ*/mm^−1^	0.804	3.491
Crystal size/mm	0.4 × 0.3 × 0.2	0.6 × 0.3 × 0.1
No. measured refl.	54765	126,491
Obs. refl. [*I* > 2*σ* (*I*)]	7188	13,660
*θ*_max_/°	27.1	26.0
R [*F* ^2^ > 2*σ*(*F*)], w*R* (*F* ^2^), *S*	0.074, 0.231, 1.09	0.054, 0.160, 1.07
Refl./param.	9985/592	17948/1084
Weighting scheme^a^	*a* = 0.1062P, *b* = 24.0229	*a* = 0.0735, *b* = 25.5728
δ*ρ*_max, min_/e × 10^−6^ pm^−3^	2.78, −1.30	1.42, −1.67

^a^ $$ W\; = \;\frac{1}{{\sigma^{2} (F_{0} )^{2} + (a \cdot P)^{2} + b \cdot P}}   {\text{where }}P = \frac{{F_{0}^{2} + 2 \cdot F_{c}^{2} }}{3} $$

The structure was solved by the Patterson method (SHELXS97 [18]). Refinement was performed by the full-matrix least-squares method based on *F* with anisotropic thermal parameters for all non-hydrogen atoms. Hydrogen atoms were inserted in calculated positions and refined riding with the corresponding atom. Four of the six crystallographic independent butoxo ligands in **1** were disordered and refined with about 50 % for each position. The same treatment was done for three of the 3-bromopropyl moieties and two isopropoxo ligands in **2**. Furthermore, one 3-bromopropyl moiety was refined using three different positions with 42, 36, and 21 % occupancy.

CCDC-1402779 (for **1**) and 1402780 (for **2**) contain the supplementary crystallographic data. These data can be obtained free of charge from the Cambridge Crystallographic Data Centre via www.ccdc.cam.ac.uk/data_request/cif.

## References

[1] Poojary DM, Bhardwaj C, Clearfield A (1995). J Mater Chem.

[2] Vermeulen LA, Fateen RZ, Robinson PD (2002). Inorg Chem.

[3] Costantino F, Sassi P, Geppi M, Taddei M (2012). Cryst Growth Des.

[4] Byrd H, Clearfield A, Poojary D, Reis KP, Thompson ME (1996). Chem Mater.

[5] Alberti G, Vivani R, Murcia Mascarós S (1998). J Mol Struct.

[6] Vivani R, Costantino F, Nocchetti M, Gatta GD (2004). J Solid State Chem.

[7] Vivani R, Costantino F, Costantino U, Nocchetti M (2006). Inorg Chem.

[8] Taddei M, Costantino F, Vivani R (2010). Inorg Chem.

[9] Taddei M, Costantino F, Manuali V, Vivani R (2011). Inorg Chem.

[10] Donnadio A, Pica M, Taddei M, Vivani R (2012). J Mater Chem.

[11] Taddei M, Vivani R, Costantino F (2013). Dalton Trans.

[12] Feichtenschlager B, Pabisch S, Peterlik H, Kickelbick G (2012). Langmuir.

[13] Lomoschitz CJ, Feichtenschlager B, Moszner N, Puchberger M, Müller K, Abele M, Kickelbick G (2011). Langmuir.

[14] Czakler M, Artner C, Schubert U (2013) Eur J Inorg Chem 5790 10.1002/ejic.201400051 PMC4362471 25814832

[15] Czakler M, Artner C, Schubert U (2014) Eur J Inorg Chem 2038 10.1002/ejic.201400051 PMC4362471 25814832

[16] Chandrasekhar V, Senapati T, Dey A, Hossain S (2011). Dalton Trans.

[17] Czakler M, Artner C, Schubert U (2014). Monatsh Chem.

[18] Sheldrick GM (1997). Program for Crystal Structure Determination.

